# Parenteral Nutrition in Palliative Cancer Care: Detrimental, Futile, or Beneficial?

**DOI:** 10.3390/curroncol31050208

**Published:** 2024-05-11

**Authors:** Erik Torbjørn Løhre, Tora Skeidsvoll Solheim, Gunnhild Jakobsen, Ola Magne Vagnildhaug, Terese Louise Schmidberger Karlsen, Ragnhild Hansdatter Habberstad, Trude Rakel Balstad, Morten Thronæs

**Affiliations:** 1 Cancer Clinic, St. Olavs Hospital, Trondheim University Hospital, 7030 Trondheim, Norwaygunnhild.jakobsen@ntnu.no (G.J.); morten.thrones@ntnu.no (M.T.); 2 Department of Clinical and Molecular Medicine, Faculty of Medicine and Health Sciences, NTNU—Norwegian University of Science and Technology, 7030 Trondheim, Norway; 3 Centre for Crisis Psychology, Faculty of Psychology, University of Bergen, 5007 Bergen, Norway; 4 Department of Public Health and Nursing, Faculty of Medicine and Health Sciences, NTNU—Norwegian University of Science and Technology, 7030 Trondheim, Norway; 5 Department of Clinical Medicine, Clinical Nutrition Research Group, UiT, The Arctic University of Norway, 9019 Tromsø, Norway

**Keywords:** parenteral nutrition, palliative cancer care, symptom burden, tolerance, nausea

## Abstract

Palliative cancer care patients may live for a long time, but malnutrition worsens the prognosis. Parenteral nutrition (PN) is suitable for replenishing a calorie deficit, but its advantages and tolerance late in the cancer trajectory are debated. We examined symptom development in hospitalized patients with and without PN. A total of 21 palliative cancer care patients receiving PN and 155 palliative cancer care patients not receiving PN during hospitalization in a specialized unit were retrospectively compared. We studied symptom intensity at admission, symptom relief during the hospital stay, and survival. The patients had locally advanced or metastatic cancer, a mean age of 70 years, and their median ECOG performance status was III. Symptom burden at admission was similar in the compared groups. Symptom relief during hospitalization was also similar. However, patients already on PN at admission reported more nausea and patients receiving PN during hospitalization reported better nausea relief compared to patients not receiving this intervention. Overall median survival was less than two months and similar in the compared groups. Based on a limited number of observations and a suboptimal study design, we were not able to demonstrate an increased symptom burden for palliative cancer care patients receiving PN late in the disease trajectory.

## 1. Introduction

Palliative care aims to relieve suffering and symptom burden in patients with life-threatening illnesses, and early palliative care interventions are recommended and beneficial [1,2]. Integration of oncology and palliative cancer care services entails early referrals, and acute palliative care units (APCUs) practicing early integration are endorsed by the European Society for Medical Oncology (ESMO) [1,3,4]. Symptom relief through the application of useful interventions is among the essential goals of integrated care, yet the most advantageous contents of a joint oncology and palliative care service are not defined [1,5].

With modern cancer treatment options, a large number of patients may be candidates for palliative care services for an extended period of time [6]. These patients need to be adequately fed to be able to benefit from long-term palliative care interventions [7]. Still, even though malnutrition worsens the prognosis, advanced nutritional interventions late in the cancer trajectory may not be beneficial [8]. One quality frontier in modern medicine is avoiding overuse and reducing non-beneficial interventions [5,9]. Thus, a scrutinizing look at advanced nutritional interventions late in the cancer trajectory is warranted.

Guidelines recommend nutritional screening and nutritional support to ensure an adequate intake in all patients receiving anticancer treatment, and to maintain nutritional status at least 25–30 kilocalories (kcals) per kilogram (kg) of body weight per day is recommended [10,11]. A decrease in the invasiveness of nutritional interventions is recommended in patients with an expected survival of less than a few months, and comfort care is recommended in patients with only a few weeks of expected survival [11]. In patients with malignant bowel obstruction (MBO), adequate nutrition by the oral or enteral route may be impossible [11]. For these patients, parenteral nutrition (PN) may improve their outcomes, despite the low-level evidence to support such a conclusion [11,12,13]. Based on available evidence, PN may benefit a limited percentage of patients with advanced cancer, but the gain is more likely in patients with good performance status and a substantial cancer-related expected survival [14,15]. For some patients with advanced cancer and malnutrition, the only result of initiating PN might be an increased risk for adverse events like infections and edema [8]. Still, a survey on the healthcare provider perspective indicated that some patients with advanced cancer appeared to benefit from PN [16]. With the agreed-upon focus on symptom relief in palliative cancer care in mind, the effects of PN on patient-reported outcomes are understudied [1,17]. For instance, nausea is prevalent in palliative cancer care patients and may be a reason for choosing PN over oral or enteral alternatives [11,18,19]. Additionally, this symptom has long been considered a side effect of PN administration in advanced cancer [20,21]. However, randomized trials comparing nausea in palliative patients receiving PN or not reported non-significant differences or results favoring PN [8,22]. Consequently, the knowledge base on patient-reported symptom development during PN treatment should be expanded.

Previously, we demonstrated comparable symptom improvements during hospitalization in an APCU for cancer patients still receiving oncological care and for patients receiving palliative care only [23]. The primary study also presented the interventions carried out during hospitalization, including medical nutrition therapy and antibiotic treatment [23]. In this secondary analysis, we studied symptom relief and remaining lifespan for patients admitted to an APCU and provided PN during the hospital stay. The primary objective was to compare symptom development from admission to discharge in patients receiving in-hospital PN and not. Secondarily, we compared infection prevalence and survival. Exploratorily, we studied PN patients with respect to care trajectory, indications, dosing, and to what degree PN was continued after discharge. 

## 2. Materials and Methods

### 2.1. Design

Results from the primary study, with a prospective longitudinal observational design and conducted at the APCU, Cancer Clinic, St. Olavs Hospital, Trondheim University Hospital, Norway, are already published [23]. The Cancer Clinic is an ESMO-labeled center of integrated oncology and palliative care. The APCU is located within the Cancer Clinic and has 12 beds, a designated staff, and approximately 450 yearly admissions. In the primary study, all patients admitted to the APCU between 15 January 2019 and 15 January 2020 were assessed. The current study comprises secondary and supplementary analyses of data from the primary study.

### 2.2. Patients

Referrals to the APCU consist of adult cancer patients with advanced disease and a need for palliative care involvement. Both patients with and without ongoing cancer treatment are admitted. Patients with hematological, gynecological, and pulmonary malignancies are treated elsewhere and only referred to the APCU when in need of neuraxial pain management [23]. Cancer treatment options, malignant alimentary tract obstructions, inadequate oral intake, and expected survival are factors taken into consideration when deciding on PN or not. The current analysis included all patients with self-reported symptom registrations available both at admission and discharge. Readmissions during the study period were excluded from the analysis.

### 2.3. Assessments

Systematic patient-reported symptom intensities indicated from 0 to 10 on the numeric rating scale (NRS 0–10) were retrieved from the primary data files to study symptom relief. The applied assessment period was past 24 hours, and registrations at admission and discharge were collected. Symptom intensity at admission and the mean change in intensity for each of the assessed symptoms during hospitalization were compared for patients receiving and not receiving PN, respectively. Physician-recorded information on patient demographics was collected. This included age, gender, cancer diagnosis, Eastern Cooperative Oncology Group (ECOG) performance status, metastatic status, and care trajectory (integrated oncology and palliative care services or palliative care only) [24]. In addition to the description of metastatic status, available information on the degree of intrabdominal cancer manifestations, including abdominal carcinomatosis and MBO, was gathered. Furthermore, alongside infection treatment during the hospital stay, available data on the use of PN, including prehospital administration, dosing during hospitalization, and use at discharge, was collected. Moreover, survival after admission was registered. 

### 2.4. Statistical Analysis

Descriptive statistics were used for demographics and clinical data.

For group comparison of symptom intensity at admission in patients receiving PN during hospitalization or not, an independent sample *t*-test was used. The normal distribution was verified by visual inspection of probability–probability (P–P) plots. Due to the limited sample size, the nonparametric Mann–Whitney U test was used for the comparison of symptom intensity at admission in patients using PN at admission or not.

A paired sample *t*-test was applied to compare symptom intensity at admission and discharge. Group differences in change in symptom intensities during hospitalization compared to patients receiving PN or not were calculated using an independent sample *t*-test. Normal distributions were verified by visual inspection of P–P plots. 

Pearson’s chi-squared test was used for the comparison of infection prevalence during hospitalization in patients receiving PN or not. 

Survival was calculated as the median days of survival from admission. The difference in survival for patients receiving PN during hospitalization or not was calculated using a log-rank test. 

*p*-values ≤ 0.05 were considered statistically significant.

SPSS statistical software (Version 29.1) (International Business Machines (IBM), Armonk, NY, USA) was applied for statistical analyses.

### 2.5. Ethics

The Regional Committee for Medical Research Ethics, Health Region Central Norway (REK) (2018/925/REK midt and 2021/212312/REK midt) defined both the primary study and the secondary analyses as healthcare improvement. Thus, according to Norwegian health care legislation, explicit informed consent from the patients was not needed.

## 3. Results

### 3.1. Demographics

A total of 195 readmissions were excluded from the 451 hospitalizations included in the primary study [23]. Out of 256 unique patients, 176 had available self-reported symptom registrations at admission and discharge and were subjected to the current analysis. A total of 21 out of 176 patients received PN during the hospital stay. Patient characteristics are described in Table 1. Overall, the mean age was 70 years, and 67% were females. The most frequent diagnoses were gastrointestinal and urological cancers. Overall median ECOG status was III (range I–IV), 88.6% had metastatic, and 11.4% had locally advanced disease. Approximately 40% of the 176 patients received integrated oncological and palliative care, and the corresponding percentage for the PN group was 38. 

### 3.2. Symptom Burden at Admission for the Compared Groups

Self-reported symptom intensities at admission for patients receiving PN during the hospital stay compared to patients not receiving this intervention are delineated in Table 2. There were no statistically significant differences in symptom intensities at admission for the 21 patients receiving PN during hospitalization compared to the 155 that were not. Nine out of the 21 patients had already received PN at admission. For those, the mean symptom score for nausea at admission (NRS 0–10, 4.4 vs. 2.1, respectively, *p* = 0.01) was significantly higher compared to the 167 patients not receiving PN at admission. Besides nausea, no statistically significant corresponding group differences were detected. 

### 3.3. Symptom Relief during Hospitalization for the Compared Groups

Self-reported symptom intensity differences from admission to discharge for patients receiving PN during hospitalization compared to patients not receiving this intervention are presented in Table 3. Patients receiving PN achieved superior symptom relief for nausea (*p* = 0.02); otherwise, there were no statistically significant differences in symptom relief during hospitalization for the two compared groups. 

### 3.4. In-Hospital Infections 

A total of 9 out of 21 (43%) patients receiving PN during hospitalization were treated for infections during the hospital stay. The corresponding fraction and percentage for patients not receiving in-hospital PN were 60 out of 155 (39%). The difference was not statistically significant (*p* = 0.72). 

#### 3.4.1. PN Indications 

For one patient, head and neck cancer-related dysphagia was decisive for the initiation of PN. Three patients had esophageal cancers, and five had gastro-duodenal tumor growth. For nine patients, MBO distal to the ligament of Treitz was decisive. In addition, two patients with nausea and one patient with an otherwise reduced general condition received PN as a supplement to their oral intake.

#### 3.4.2. PN Dosing 

All patients receiving PN during hospitalization were treated with SmofKabiven^®^ (Fresenius, Kabi), which is a solution suitable for total PN. For the patients receiving a stable dose during the hospital stay, the mean PN dose corresponded to 19.1 kcals/kg/day (range 6.2–33.3). Only three patients had no oral intake. 

#### 3.4.3. PN Continuation at Discharge

A total of 11 out of 21 patients continued PN at discharge. In eight patients, PN was discontinued at discharge, and two died during the hospital stay. Out of the nine patients already using PN at admission, six continued PN at discharge, and one died during the hospital stay.

### 3.5. Survival after Admission

Median (95% confidence interval (CI)) survival after admission for patients receiving in-hospital PN was 48 (41–55) days. The corresponding survival for patients not receiving in-hospital PN was 58 (44–72) days. The survival difference was not statistically significant (*p* = 0.5). 

## 4. Discussion

### 4.1. Statement of Principal Findings

We demonstrated that palliative cancer care patients administered PN during hospitalization in an APCU achieved symptom relief comparable to those not treated with PN. The fraction of patients receiving integrated oncology and palliative care services was similar in the compared groups. Patients already on PN at admission reported more nausea, whereas patients receiving PN during hospitalization reported more improvement in nausea compared to patients not receiving PN. There were no group differences in infection prevalence. In addition, most patients received PN doses lower than their calorie requirements. Furthermore, there were no survival differences between patients who received PN during hospitalization and those who did not. 

### 4.2. Appraisal of Methods

Retrospective analyses have inherent design limitations, are prone to bias, and are unable to demonstrate cause–effect relationships [25]. Even though the results may serve a hypothesis-generating purpose, the generalization of the results should be conducted with the utmost caution [25]. This study is a secondary analysis, and the primary study was designed to describe medical interventions and symptom management in an APCU [23]. Therefore, the analyzed data were collected for reasons other than the study objectives [26]. In addition, the small sample size and study context represent further limitations to this single-institution retrospective secondary analysis and may result in false positive results, overestimation of the magnitude of associations, and limited external validity [27,28]. 

### 4.3. Comparison with Previous Work

#### 4.3.1. PN and Symptom Burden 

A randomized controlled trial (RCT) comparing PN and oral feeding in patients with advanced cancer and a short life expectancy reported significantly more pain but insignificantly more nausea at one month in patients receiving PN [8]. For patients with incurable cancer and longer expected survival, another RCT showed improved quality of life (QoL) after twelve weeks of home PN treatment compared to best-practice nutritional care and dietetic counseling [22]. In an expert Delphi survey, QoL was ranked as the most important quality of care indicator for long-term PN treatment in cancer patients [29]. Later, two non-randomized studies reported improved QoL in cancer patients after one and three months of PN treatment, respectively [30,31]. For patients with advanced cancer and limited expected survival, both the expected benefits and burdens of nutritional interventions should be carefully discussed with the patient [10]. Based on limited data, we were unable to demonstrate unfavorable symptom intensity development during hospitalization for patients with advanced cancer, a short life expectancy, and treated with PN. The reported nausea improvement in patients receiving PN was of interest, as was the finding of increased nausea in patients already on PN at admission. Plausible explanations for these seeming contradictions include nausea as a contributing factor for PN initiation, PN-induced nausea, home management of nausea, and a specific focus on nausea management during hospitalization in an APCU. In addition, differences in the distribution of diagnoses may have contributed (Table 1). Methodological considerations include a larger potential for improvement in higher symptom scores and a limited sample size. 

#### 4.3.2. PN and Complications

PN may result in a variety of treatment-related complications, like hyperglycemia, refeeding syndrome, thrombosis, and serious infections [32]. We previously demonstrated that approximately one-quarter of the patients acutely admitted to the APCU received intravenous antibiotics [33]. Taking the inherent limitations of a short observation period and a restricted number of patients into account, we were not able to detect any difference in infection prevalence for hospitalized patients treated with PN compared to patients not receiving this intervention. However, the fact that PN was discontinued in eight patients at discharge might be due to both reflections on limited expected benefits and potential complications. 

#### 4.3.3. PN and Indications

Reports on the frequency of PN administration in palliative cancer care patients have been previously published [34,35]. One paper reported that 13% of patients in palliative care units received PN and hydration [34], and another that 11% of patients in urban home care units received PN [35]. In our study, the corresponding percentage was approximately 12. For selected cancer patients with MBO, PN may be a relevant option [36]. However, a prematurely closed randomized trial was unable to determine the role of PN in patients with advanced cancer and non-functioning bowels [37]. Still, a later systematic review stated that QoL and physical function may improve during cancer treatment in PN patients unable to feed enterally [17]. PN is considered supplemental in patients with maintained oral intake [7]. Supplemental PN (SPN) may be relevant for many patient categories, including cancer patients at risk of dying rather from starvation than from tumor progression [7,38]. The majority of our patients had mechanical obstructions, yet some had oral intake. 

#### 4.3.4. PN and Dosing

With an estimated total energy expenditure in cancer patients of at least 25 kcal/kg/day, the registered mean dose of approximately 19 kcal/kg/day in the present study is below nutritional requirements [10]. However, as only three patients had no oral intake, for most of our patients, parenteral feeding in practice represented SPN. Additionally, clinical decisions on PN dosing are guided both by requirements and potential side effects. Thus, the doses provided in the current study may also reflect clinical evaluations of PN tolerability.

### 4.4. Implications and Further Work

Sample size and study design preclude extensive interpretations and definitive implications of the current secondary analysis. Still, the results may serve as a basis for the hypothesis that PN may not necessarily be detrimental to patients with advanced cancer. Evidence on the effects of PN in this group of patients, however, must be collected from larger studies with improved design. Further studies of the potential benefits of SPN earlier in the cancer trajectory may also be warranted.

## 5. Conclusions

Based on a restricted number of patient observations and a study design allowing limited inferences, we were not able to demonstrate PN-related increased symptom burden for hospitalized cancer patients late in the disease trajectory.

## Figures and Tables

**Table 1:** Demographic and clinical characteristics.

Characteristic	Parenteral Nutrition (*n* = 21) *n*	%	Oral or Enteral Nutrition (*n* = 155) *n*	%
Age, years, mean (SD ^1^)	68 (12)		71 (14)	
Gender				
Female	6	28.6	53	34.2
Male	15	71.4	102	65.8
Cancer diagnosis				
Gastrointestinal	16	76.2	60	38.7
Urological	2	9.5	42	27.1
Breast	1	4.8	11	7.1
Head and neck	1	4.8	12	7.7
Other	1	4.8	29	18.7
Missing	0	0	1	0.6
Metastases				
Skeletal	1	4.8	68	43.9
Liver	8	38.1	58	37.4
Lung	1	4.8	43	27.7
Nodes	7	33.3	53	34.2
Brain	0	0	11	7.1
Abdominal carcinomatosis	9	42.9	30	19.4
Comorbidity, present/yes	16	76.2	115	74.2
Performance status				
ECOG ^2^ I–II	6	28.6	79	51.0
ECOG ^2^ III–IV	15	71.4	76	49.0
Care trajectory				
Integrated oncology and palliative	8	38.1	63	40.6
Palliative	13	61.9	91	58.7
Missing	0	0	1	0.6
Days alive after admission, median	48 (41–55)		58 (44–72)	
(95% CI ^3^)				

^1^ Standard deviation, ^2^ Eastern Cooperative Oncology Group, ^3^ Confidence interval.

**Table 2:** Patient-reported symptom intensities at admission for patients receiving/not receiving PN during hospitalization.

Symptom	Mean NRS Score ^1^			SD ^2^
	Parenteral Nutrition Mean (SD)	Oral or Enteral Nutrition Mean (SD)	Difference ^3^	*p*-Value
Average pain	3.4 (2.5)	3.9 (2.6)	−0.5	0.41
Tiredness	6.2 (1.9)	5.5 (2.5)	0.7	0.19
Drowsiness	5.8 (1.9)	5.0 (2.4)	0.8	0.15
Nausea	2.8 (2.8)	2.1 (2.5)	0.7	0.22
Appetite	5.6 (3.2)	4.5 (3.1)	1.1	0.17
Shortness of breath	3.4 (2.8)	3.3 (2.7)	0.1	0.79
Depression	3.2 (2.3)	3.5 (2.7)	−0.3	0.73
Anxiety	2.6 (2.3)	3.0 (2.6)	−0.4	0.57
Wellbeing	4.2 (2.1)	4.5 (2.3)	−0.3	0.51
Constipation	3.1 (4.0)	3.0 (3.1)	0.1	0.92
Sleep	4.6 (2.6)	4.0 (2.8)	0.6	0.32
Worst pain	4.9 (2.9)	5.2 (3.0)	−0.3	0.69

^1^ Cancer-related symptoms assessed on a 0–10 NRS at admission. Scores: 0 = best, 10 = worst. ^2^ Standard deviation. ^3^ A positive difference indicates numerically higher symptom intensity in the parenteral nutrition group.

**Table 3:** Reduction in patient-reported symptom intensities during hospitalization.

Symptom ^1^	Mean NRS Score ^1^			SD ^2^
	Parenteral Nutrition Mean (SD)	Oral or Enteral Nutrition Mean (SD)	Difference ^3^	*p*-Value
Average pain	0.9 (2.5)	1.1 (2.4)	−0.2	0.63
Tiredness	1.9 (2.1)	1.1 (2.3)	0.8	0.17
Drowsiness	1.8 (2.1)	0.9 (2.5)	0.9	0.14
Nausea	1.8 (2.8)	0.6 (1.9)	1.2	0.02
Appetite	0.9 (4.4)	1.0 (2.5)	−0.1	0.87
Shortness of breath	1.1 (1.6)	0.8 (2.2)	0.3	0.66
Depression	0.6 (2.0)	0.5 (2.3)	0.1	0.92
Anxiety	1.1 (2.1)	0.6 (2.2)	0.5	0.30
Wellbeing	0.5 (3.6)	1.1 (2.4)	−0.6	0.39
Constipation	1.7 (2.8)	1.1 (3.0)	0.6	0.45
Sleep	1.3 (4.0)	0.7 (2.9)	0.6	0.51
Worst pain	1.5 (2.7)	1.6 (2.8)	−0.1	0.88

^1^ Cancer-related symptoms were assessed on a 0–10 NRS at admission and discharge. Scores: 0 = best, 10 = worst. ^2^ Standard deviation. ^3^ A positive difference indicates numerically more improvement in the parenteral nutrition group.

## Data Availability

The data presented in this study are available on request from the corresponding author.
